# Translation, cross-cultural adaptation, and psychometric properties of the Brazilian Portuguese version of the Consumer Financial Protection Bureau Financial Well-Being scale

**DOI:** 10.47626/2237-6089-2020-0034

**Published:** 2021-01-22

**Authors:** Anna Beatriz Carnielli Howat-Rodrigues, Jerson Laks, Valeska Marinho

**Affiliations:** 1 Universidade Federal do Rio de Janeiro Instituto de Psiquiatria Rio de JaneiroRJ Brazil Instituto de Psiquiatria (IPUB), Universidade Federal do Rio de Janeiro (UFRJ), Rio de Janeiro, RJ, Brazil.

**Keywords:** Financial well-being, surveys and questionnaires, cross-cultural, validation studies

## Abstract

**Objective:**

To translate and back-translate the Consumer Financial Protection Bureau (CFPB) Financial Well-Being Scale into Brazilian Portuguese, to assess its cross-cultural semantic equivalence, and to verify the psychometric properties of the final version.

**Methods:**

Adaptation of the original scale applied a three-step methodology: translation and back-translation, appreciation of semantic equivalence, and administration to a convenience sample of 834 subjects. The analysis of psychometric properties comprised evaluation of evidence of the instrument’s validity by factor analysis, validity by contrasting groups, and internal consistency with Cronbach’s alpha coefficient. The CFPB granted authorization to conduct cross-cultural adaptation into Brazilian Portuguese.

**Results:**

Results indicated adequate cultural adaptation between scales, with good equivalence between the original English version and the final Brazilian version. The Cronbach’s alpha coefficient for the instrument’s internal consistency in this sample was 0.89. Exploratory and confirmatory factor analyses demonstrated high levels of item reliability and goodness of fit, with all 10 items loading onto a single factor, financial well-being. The measure has shown structural stability in two different cultural contexts (Brazil and the USA).

**Conclusion:**

The Brazilian version demonstrated acceptable psychometric properties and adequate structural and cross-cultural validity and the participants found it easy to understand.

## Introduction

Financial well-being is defined as a state of being financially healthy, happy, and free from financial worries. ^1^ From the perspective of consumers, financial well-being can be described as a feeling of being able to fully meet current and ongoing financial obligations, feeling secure about the financial future, and being able to make choices to enjoy life. ^1^^-^^4^

An individual’s perspective on financial well-being is related to several variables such as personality characteristics, life experiences, behavior, concern, and personal judgment about the common personal finance topics of money, credit, and economic resources. ^3^ Furthermore, there is general agreement that financial empowerment plays an important role in economic security and the sense of self-sufficiency, with positive impacts on quality of life (QoL), health, and well-being. ^4^^-^^6^

The interface between psychology and economics has become a prominent field of research and is concerned with both the psychological basis of the economic behaviors of individuals and also the impacts of economic issues on individuals’ psychology. For instance, data have shown that financial strains and negative reaction to an adverse economic condition is associated with reduced psychological well-being, ^7^ and mental disorders such as depression and anxiety among people who are over-indebted. ^8^ For this reason, specific measures of financial well-being and financial satisfaction are crucial for economic psychology.

The Consumer Financial Protection Bureau (CFPB) Financial Well-Being Scale ^1^^,^^2^^,^^9^ is a 10-item instrument that measures consumer-driven financial well-being. The CFPB measures and quantifies a subjective aspect by comparing it over time and/or to other variables of interest. It quantifies the financial situation and the financial capacity developed throughout life and provides a measure of perceptions of security and freedom of choice. The scale was constructed based on strong psychometric properties, and by applying measurement theory analyses including factor analysis and Item Response Theory (IRT) modeling. The scale is an easy-to-use measure across different populations in many research undertakings and population settings. Data gathered from qualitative studies suggest that age can pose differences in the way people measure financial health. As such, data were collected and analyzed in two separate samples - older consumers (aged 62 and older) and younger consumers (aged 18-61). The authors have shown that the scale is one-dimensional with Cronbach’s alpha coefficients of 0.89 and 0.90 (Online administration, age 18-61 and age 62+, respectively). ^9^

Objective measures of financial well-being are not yet available in Brazilian Portuguese. Financial education is key to promoting positive financial behaviors and to achieving better financial satisfaction and well-being. For these reasons, understanding, measuring, and promoting financial well-being are important steps in promoting health and QoL. Brazil is a middle-income country that still needs to develop a culture of investing and saving as a means of achieving higher levels of welfare and satisfaction.

The main objectives of this study were to translate and back-translate the CFPB financial well-being scale into Brazilian Portuguese, to assess its cross-cultural semantic equivalence, and to verify the psychometric properties of the final version. As a secondary objective, the study analyzed the power of the Financial Well-Being scale to differentiate participants with regard to purchasing power and QoL, since it was expected that groups with higher levels of financial well-being would exhibit higher scores for purchasing power and QoL.

## Methods

The CFPB Financial Well-Being instrument is a 10-item 5-point Likert scale, extensively tested and validated to ensure validity and reliability. The scale is driven by the consumer’s perspective of their financial well-being and yields a single score, which captures its core elements, namely, control over one’s finances, the capacity to absorb a financial shock, being on track to meet financial goals, and the capacity to make choices that allow one to enjoy life. ^1^^,^^2^^,^^9^ Each of the 10 items is rated according to a Likert label representing opinions and attitudes concerning specific financial topics: completely; very well; somewhat; very little; not at all (items 1-6), or always; often; sometimes; rarely; never (items 7-10).

Scale final scores enable both comparisons between individuals and tracking of the same individual’s changes over time. The CFPB Financial Well-Being Scale is scored based on an Item Response Theory (IRT) analysis, a statistical method that provides a more precise individual estimate that allows people’s responses to different items to contribute accordingly to the final score. ^9^ Determining the final score is a two-step process. The first step aims to determine the total response value or raw total. The total value is obtained by adding up each person’s responses (from 0 to 4) to the individual items. This total response value will be used in step 2, in which it is converted into the CFPB Financial Well-Being Scale score. The final score incorporates respondent’s age group, regardless of whether the questionnaire was self-administered or administered by someone else. The items allow a single score to be generated by measuring the core elements of financial well-being. ^1^^,^^2^^,^^9^

### Ethics

This study was approved by the Human Research Ethics Committee at the Instituto de Psiquiatria (IPUB) at the Universidade Federal do Rio de Janeiro (UFRJ) (CAAE 58096816.0.0000.5263) and is part of a larger study evaluating Financial Risk and Well-Being in Investors’ Decision-Making. All participants signed an informed consent statement before entering the study group.

### Translation and item adaptation of the CFPB well-being scale

The following consecutive steps were implemented: initial translations, synthesis of translations, back-translation, review of semantic equivalence by an evaluation committee, and pretesting of the final version. ^10^

### Psychometric and discriminating properties

A final version, adjusted according to feedback provided by participants of the convenience sample, was administered to 834 subjects on the online mailing list of the Brazilian Securities and Exchange Commission (264 females) who responded to an invitation. Mean age of participants was 39.27 (standard deviation [SD] = 10.82), they had high educational level (60.9% post-graduate), were married or living together (60%), and pertained to a group of Brazilians in economic classes A to B according to the Brazilian Economic Classification Scores Criteria (CCEB; mean [M] = 41.36, SD = 13.27). ^11^

To achieve one of the main study objectives and verify the psychometric properties and test reliability of the final version, Cronbach’s alpha reliability index was used to test the validity of the scale’s internal structure and exploratory and confirmatory analyses were performed to test the structural validity of the measure. Evidence of validity based on external criteria was acquired by analyzing contrasting groups.

### Instruments

Sociodemographic measures and local criteria for economic classification were collected (gender, age, academic background, and Brazilian Economic Classification Score Criteria-CCEB). ^11^ The original CCEB has a categorical format and provides information on economic classes ranging from Class A to Class E according to a points system, as follows, economic Class A: 45 - 100 points, B1: 38-44; B2: 29-37; C1: 23-28; C2: 17-22; and D-E: 1-16. In order to meet this study’s aims, the original purchasing power index scores were used as a quantitative variable (range: 0-100 points).

Purchasing power in Brazil is established by the Brazilian Association of Market Research Companies ^11^ based on a yearly survey conducted by the Brazilian Institute of Public Opinion and Statistics (IBOPE) of social, demographic and economic data on Brazilian families in metropolitan regions. It is an index used to classify the population into socioeconomic classes. In this study we used this index as a proxy for income. ^12^ QoL was assessed with a 5-point Likert response scale for the question “How would you score your current quality of life?”.

### Data analysis

Data were analyzed using the Statistical Package for Social Sciences (SPSS), version 16.0 and Analysis of Moment Structures (Amos), version 7.0. A descriptive analysis was performed with all the scale items, and the database was randomly re-sampled (using the SPSS function for selecting random samples of cases) into two databases of the same size (approximately 50% of cases were allocated to each of the two database). The dimensional structure of the instrument was verified in one subset of the sample using exploratory factor analysis (EFA). Cronbach’s alpha reliability indexes were computed for the items of the resulting subscales. Confirmatory factor analysis was then conducted with the second subset from the database, which also computed the reliability coefficients of the resulting subscales.

Evidence of the validity of the internal structure was assessed based on both exploratory factor analysis and confirmatory factor analysis results. We also verified the validity of measures by analyzing contrasting groups. For the analysis of contrasting groups, we used differentiation based on quartiles of the original well-being score, and tested whether these groups were different in terms of QoL and purchasing power.

The Maximum likelihood (ML) estimation method was used to test the goodness of fit of the models proposed by CFA. According to Byrne’s suggestions, ^10^ the following indexes were analyzed for confirmatory factor analysis (CFA): 1) χ^2^ (chi-square) – model fit index (values lower than 5 – not significant – are recommended); 2) χ^2^/df – fit indicator (values between 2 and 5 are recommended); 3) comparative fit index (CFI) – a comparative indicator of the model fit (values greater than 0.9 are recommended); 4) root mean square error of approximation (RMSEA) - index of residual suitability for confirmatory strategies with large samples (values lower than 0.08 with a 90% confidence interval are recommended).

Evidence of validity based on external criteria was assessed using QoL and purchasing power scores. Original scores from the CFPB financial well-being scale were sliced into quartiles and the resulting subsets were cross-compared in terms of their QoL and purchasing power. Multivariate analysis of variance (MANOVA) (n = 834) was used for the comparative analysis between these groups. The d statistic was used to estimate the effect size of Cohen’s d, which is given in the form of the percentage of SD. The higher d is, the stronger the effect of association between the pairs tested. ^13^

## Results

The results from the translation and cross-cultural adaptation followed the steps mentioned above. The first step consisted of translating the original instrument into Brazilian Portuguese. Two independent native Portuguese speakers who were qualified professionals fluent in English translated the 10 items of the CFPB well-being scale into Brazilian Portuguese in this first step. For seven items (2, 3, 5-8, and 10) there was 100% concordance. For the remaining three items (1, 4, 9), discordance was 33.3%, in these cases, the items that were in the final version were those that were translated by two evaluators alike. The Likert response options were also translated with 100% concordance. A synthesis version was then constructed. Step 2 consisted of backward translations by an independent qualified professional whose native language was English.

To improve the translation’s semantic properties and its appropriateness in Portuguese, all items were presented to a convenience sample comprising 10 individuals (both genders, mean age of 43.3, age range 21-63) attending a conference organized by the Brazilian Securities and Exchange Commission. Clarity and objectivity were analyzed and a final version for Brazilian Portuguese was subsequently constructed and presented to the convenience sample, resulting in a final version of the scale.

Scoring procedures followed the original guidance, according to a Likert model. The item scores in the Brazilian version range from 1 to 5 (from 1 representing “not at all” or “never” to 5 representing “completely” or “always”). To generate the final score, items 1, 2, 4, and 8 are inverted and added to the remaining items. As a result, the Brazilian version’s scores are higher when financial well-being is worse. Initially, the main scale components were analyzed to verify the adequacy of the data matrix for the factorial analysis procedure and to determine the number of factors to be extracted. This step resulted in a KMO of 0.93 and Bartlett’s test of sphericity was significant at 1921.72, df = 45, p < 0.001. These values satisfy the conditions for the factorial procedure.

Cattell’s scree plot criterion and elements related to theoretical interpretation of the construct were used to decide on the number of factors to be extracted. The scree plot suggested a single factor solution, which explained 52.80% of variance.

Since extraction of only one factor is not rotated and the number of factors had been defined, we used the principal axis factoring method for factor extraction and reliability calculations. Table 1 shows the results of principal axis factoring for the ten items, with communalities, variance explained, and Cronbach’s alpha reliability index.

**Table 1:** Extracted factor, factor loadings, communalities (h^2^), number of items, variance explained, and reliability index (Cronbach’s alpha) for the CFPB Financial Well-Being Scale, by the principal axis factoring method

Original version	Well-being factor	h^2^
1. I could handle a major unexpected expense	0.81	0.66
2. I am securing my financial future	0.79	0.63
3. Because of my money situation, I feel like I will never have the things I want in life	0.77	0.59
4. I can enjoy life because of the way I’m managing my money	0.76	0.58
5. I am just getting by financially	0.70	0.50
6. I am concerned that the money I have or will save won’t last	0.68	0.46
7. Giving a gift for a wedding, birthday or other occasion would put a strain on my finances for the month	0.67	0.45
8. I have money left over at the end of the month	0.64	0.41
9. I am behind with my finances	0.55	0.30
10. My finances control my life	0.47	0.22
Variance (%)	47.94	
Cronbach’s alpha	0.90	

CFPB = Consumer Financial Protection Bureau.

Aiming to verify the measure’s structural stability and to verify the one-dimensionality of the Financial Well-Being construct in the Brazilian sample, all ten items from the second database (n = 417) were loaded into the confirmatory factor analysis (CFA) using the Maximum Likelihood method. The model to be tested was the matrix that resulted from the exploratory analysis and from previous studies. ^9^

Validity by contrasting groups was tested in order to investigate the power of the financial well-being construct to differentiate participants by purchasing power and QoL. A MANOVA test was conducted with four groups classified by financial well-being scores. Group 1 had scores from 10 to 19 (n = 252); Group 2 had scores from 20 to 29 (n = 364); Group 3 had scores from 30 to 39 (n = 160); and Group 4 had scores from 40 to 50 (n = 58). Higher scores represent lower financial well-being.

All financial well-being groups were significantly different, *F* (6, 1658) = 43.26, p < 0.001, Λ = 0.75, both for purchasing power, *F* (3, 830) = 25.58, p < 0.001, and for QoL, *F* (3, 830) = 84.01, p < 0.001. The instrument’s ability to discriminate between groups was therefore demonstrated. Mean purchasing power for each group was, respectively: 42.87 (SD = 11.48), 39.18 (SD = 9.71), 36.05 (SD = 9.83), and 33.14 (SD = 12.06). Cohen’s d for purchasing power for group pairs was, successively: 0.35 (between groups 1 and 2), 0.64 (between groups 1 and 3), 0.82 (between groups 1 and 4), 0.32 (between groups 2 and 3), 0.55 (between groups 2 and 4), and 0.27. (between groups 3 and 4). Mean QoL scores for each group were, respectively: 4.54 (0.69), 4.17 (0.70), 3.63 (0.80), and 3.26 (0.97). Cohen’s d for QoL for group pairs was, successively: 0.57, 1.3, 1.59, 0.73, 1.08, and 0.41. Four of the effect sizes calculated indicated strong associations (d ≥ 0.8), seven indicated average associations (d ≥ 0.3) and only one indicated a weak association, with d = 0.27.

The goodness of fit of the initial hypothetical model was satisfactory for the original structure χ^2^ = 138.64, df = 35 (p < 0.001), χ^2^/df = 3.96, CFI = 0.94, RMSEA = 0.08, 90% CI [0.07, 0.09]. χ^2^ indexes were high and significant. According to Byrne, ^13^ these values indicate a lack of model fit. However, the same author claims that other indexes should be analyzed to make the final decision on model fit: if the χ^2^ value is significant, it should be divided by the degrees of freedom. The model is acceptable when this value is less than or equal to 5, as was found. Goodness of fit (χ^2^/df) was therefore acceptable. The CFI was satisfactory, as was RMSEA. The Cronbach’s alpha coefficient for this sample was 0.89.

**Figure 1:**
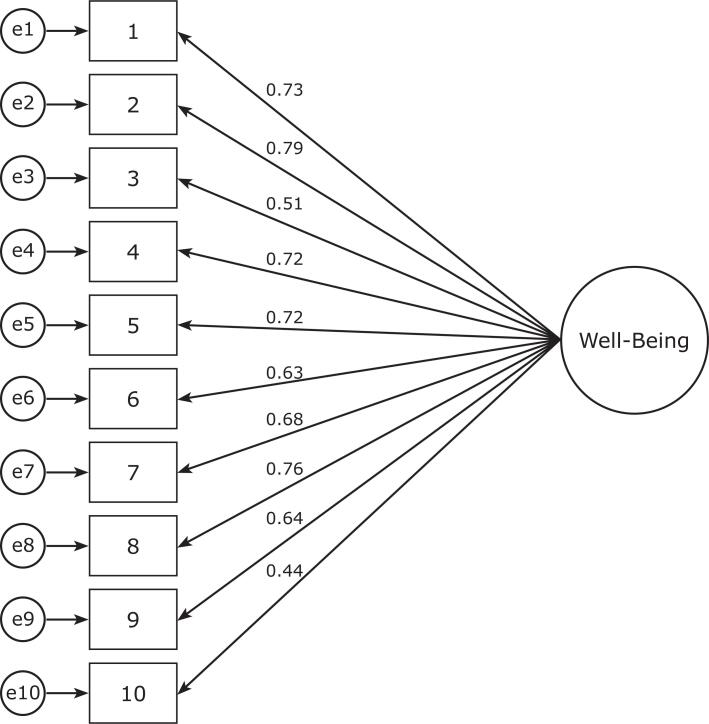
One-dimensional model of the Financial Well-Being Scale, obtained from a sample of 411 participants. Standardized estimates: regression coefficients close to unidirectional arrows and squared multiple correlations close to the variables.

## Discussion

The CFPB Financial Well-Being Scale is a reliable, public-domain tool that can be used to measure financial well-being, not only across different individuals, but also of the same individual over time. ^9^ The scale was developed using large samples and validated across different related concepts such as financial satisfaction, credit scores, experiencing economic shocks, and material hardship. Positive and significant statistical relationships were found between CFPB Financial Well-Being Scale scores and self-assessed credit quality and measures of financial resilience. It was negatively correlated with indebtedness and to the likelihood of having experienced economic hardship in the previous year. The scores also correlated with income and education in the expected directions. Higher income (M = 62.95, SD = 48.01, p = 0.38) and higher educational level (M = 3.71, SD = 1.42, p = 0.19) correlated with better financial well-being scores. ^9^

In common with the original CFPB report, ^9^ our results with the Brazilian sample indicate a one-dimensional measure with satisfactory validity and reliability. Evidence of the validity of the internal structure was assessed based on exploratory and confirmatory factor analysis. Both exploratory and confirmatory analysis showed high levels of item reliability and demonstrated goodness of fit. Additionally, in line with the original CFPB research, ^1^^,^^2^^,^^9^ the scale structure showed 10 items grouped onto one factor, termed financial well-being.

The measure has shown structural stability in two different cultural contexts (Brazil and the USA). We also verified the validity of measures analyzing contrasting groups. We expected that groups with higher levels of financial well-being would exhibit higher scores for purchasing power and QoL. ^4^^,^^5^ Our data showed the instrument’s ability to discriminate between groups: the group with higher financial well-being presented higher average purchasing power and QoL; while the group with lower financial well-being presented lower average purchasing power and QoL. This relationship was also found in the two intermediate groups. As shown in a report on older adults in the UK, also linking financial satisfaction with QoL, ^14^ our data from a middle-income country also pointed in the same direction, suggesting that improving individuals’ sense of financial security may enhance QoL.

In the Brazilian version of the scale, scores are higher when financial well-being is worse, but this does not cause differences in interpretation of the data. The present research has some limitations that should be addressed. First, the scale was administered to a convenience sample of investors and other people who have access to computers and are familiar with financial terminology. Also, the sample was unbalanced. This sample overrepresented highly educated, high-income males. It should be noted that there is another scale called the InCharge Financial Distress/Financial Well-Being Scale ^3^ considered an objective instrument used in well-being research. It is a single-factor, 8-item Likert scale. ^3^ However, the CFPB Financial Well Being Scale is a free-of-charge and instrument that is widely available.

Additionally, taking into account the heterogeneity of the Brazilian population, our findings cannot be extrapolated to the whole Brazilian population. Similar studies should be conducted to corroborate our data and gather more information in different Brazilian cities and regions.

The increasing size of the economically active population in developing countries such as Brazil, the possibility of changes in local labor laws, and the progressive increase in the purchasing power of the lower-middle income classes point to the need to improve a culture of investing and to provide financial education in our country. Since achieving financial well-being is one of the many objectives of promoting financial education, provision of adequate measures of the construct in Brazilian Portuguese is an important step. To the best of our knowledge, this is the first adapted and validated measure of financial well-being in Brazilian Portuguese and the first to demonstrate its relation to QoL and purchasing power in Brazil.

## References

[1] 1. Consumer Financial Protection Bureau. Financial well being: the goal of financial education [internet]. 2015 Jan [Cited 2020 Jul 22]. files.consumerfinance.gov/f/201501_cfpb_report_financial-well-being.pdf

[2] 2. Consumer Financial Protection Bureau. Measuring financial well-being: a guide to using the CFPB financial well-being scale [internet]. [Cited 2020 Jul 22]. www.consumerfinance.gov/data-research/research-reports/financial-well-being-scale/

[3] 3. Prawitz A, Garman ET, Sorhaindo B, O’Neill B, Kim J, Drentea P. Incharge financial distress/financial well-being scale: development, administration, and score interpretation. J Financ Couns Plan. 2006;17:34-50.

[4] 4. Zyphur MJ, Li WD, Zhang Z, Arvey RD, Barsky AP. Income, personality, and subjective financial well-being: the role of gender in their genetic and environmental relationships. Front Psychol. 2015;6:1493. 10.3389/fpsyg.2015.01493 PMC4587091 26483742

[5] 5. Gutter M, Copur Z. Financial behaviors and financial well-being of college students: evidence from a national survey. J Fam Econ Issues. 2011;32:699-714.

[6] 6. Henchoz Y, Botrugno F, Cornaz S, Büla C, Charef S, Santos-Eggimann B, et al. Determinants of quality of life in community-dwelling older adults: comparing three cut-offs on the excellent-to-poor spectrum. Qual Life Res. 2017;26:283-9. 10.1007/s11136-016-1394-3 27558783

[7] 7. Mills R, Grasmick H, Morgan C, Wenk D. The effects of gender, family satisfaction, and economic strain on psychological well-being. Fam Relat. 1992;41:440-5.

[8] 8. Holmgren R, Nilsson Sundström E, Levinsson H, Ahlström R. Coping and financial strain as predictors of mental illness in over- indebted individuals in Sweden. Scand J Psychol. 2019;60:50-8. 10.1111/sjop.12511 30585328

[9] 9. Consumer Financial Protection Bureau. Financial Well-Being Scale: scale development technical report [internet]. [Cited 2020 July 22]. www.consumerfinance.gov/data-research/research-reports/financial-well-being-technical-report/

[10] 10. Beaton DE, Bombardier C, Guillemin F, Ferraz MB. Guidelines for the process of cross-cultural adaptation of self-report measures. Spine. 2000;25:3186-91. 10.1097/00007632-200012150-00014 11124735

[11] 11. Associação Brasileira de Empresas de Pesquisa (ABEP). Brazilian Criteria 2015 and social class distribution update for 2016 [internet]. [Cited 2020 July 22]. www.abep.org/criterio-brasil

[12] 12. Howat-Rodrigues AB, Tokumaru RS. Scale of family unpredictability during childhood: validity evidence. Paidéia (Ribeirão Preto). 2014;24:11-20.

[13] 13. Byrne BM. Structural equation modeling with Mplus. New York: Routledge; 2012.

[14] 14. Collard S, Hayes D. Financial wellbeing in later life: evidence and policy. London: ILC-UK; 2014 Mar [cited 2020 Mar 24]. www.bristol.ac.uk/media-library/sites/geography/migrated/documents/pfrc1402.pdf

